# Vital signs, clinical rules, and gut feeling: an observational study among patients with fever

**DOI:** 10.3399/BJGPO.2021.0125

**Published:** 2021-11-24

**Authors:** Gideon HP Latten, Lieke Claassen, Lucinda Coumans, Vera Goedemondt, Calvin Brouwer, Jean WM Muris, Jochen WL Cals, Patricia M Stassen

**Affiliations:** 1 Emergency Department, Zuyderland Medisch Centrum, Heerlen, The Netherlands; 2 Department of Family Medicine, Maastricht University, Care and Public Health Research Institute (CAPHRI), Maastricht, The Netherlands; 3 Department of Internal Medicine, Division General Medicine, Section Acute Medicine, Maastricht University, CAPHRI, Maastricht, The Netherlands

**Keywords:** infectious illness, hospital referrals, vital signs, emergency service, hospital, general practitioners

## Abstract

**Background:**

GPs decide which patients with fever need referral to the emergency department (ED). Vital signs, clinical rules, and gut feeling can influence this critical management decision.

**Aim:**

To investigate which vital signs are measured by GPs, and whether referral is associated with vital signs, clinical rules, or gut feeling.

**Design & setting:**

Prospective observational study at two out-of-hours (OOH) GP cooperatives in the Netherlands.

**Method:**

During two 9-day periods, GPs performed their regular work-up in patients aged ≥18 years with fever (≥38.0°C). Subsequently, researchers measured missing vital signs for completion of the systemic inflammatory response syndrome (SIRS) criteria and the quick Sequential Organ Failure Assessment (qSOFA) score. Associations between the number of referrals, positive SIRS and qSOFA scores, and GPs’ gut feelings were investigated.

**Results:**

GPs measured and recorded all vital signs required for SIRS criteria and qSOFA score calculations in 24 of 108 (22.2%) assessed patients, and referred 45 (41.7%) to the ED. Higher respiratory rates, temperatures, clinical rules, and gut feeling were associated with referral. During 7-day follow-up, nine (14.3%) of 63 patients who were initially not referred were admitted to hospital.

**Conclusion:**

GPs measured and recorded all vital signs for SIRS criteria and qSOFA score in one-in-five patients with fever, and referred half of 63 patients who were SIRS-positive and almost all of 22 patients who were qSOFA-positive. Some vital signs and gut feeling were associated with referral, but none were consistently present in all patients who were referred. The vast majority of patients who were not initially referred remained at home during follow-up.

## How this fits in

When deciding whether or not to refer a patient with an infection to the hospital, GPs can use a patient’s general appearance and vital signs, gut feeling, and medical history. It is not known how often GPs measure and record vital signs in patients with fever and whether (determining) vital signs, clinical rules, or gut feeling are related to referral. In this study, GPs measured and recorded a complete set of vital signs in one-in-five patients with fever during OOH. Although associations between certain vital signs, clinical rules, and gut feeling and ED referral were found, the decision to refer a patient is probably multifactorial. To be able to further investigate this topic, vital signs should be measured and recorded consistently in all patients with fever.

## Introduction

GPs have the difficult task of deciding whether or not to refer a patient with fever to the ED. This decision can be critical, as delay in the treatment of those with a serious infection, such as sepsis, is associated with increased morbidity and mortality.^1,2^ Unfortunately, there is no gold standard for diagnosing sepsis, which makes adequate patient selection for ED referral difficult.

Professionals are encouraged to measure vital signs, in order to pick up potential signs of organ failure and diagnose sepsis.^3,4^ Two clinical rules have been developed to aid in the detection of sepsis: the SIRS criteria and the qSOFA score.^3,5^ These simple rules are often recommended as screening tools for sepsis, but it is unknown whether GPs actually measure the vital signs required for calculation of these scores.

In a retrospective survey-based study, GPs indicated that they measure vital signs in the majority of patients with a possible serious infection.^6^ However, for making the decision to refer these patients, vital signs were not considered as important as general appearance, gut feeling, and medical history of the patient. Gut feeling may be especially important, as it has been shown to play a considerable role in the diagnostic reasoning of GPs.^7,8^ To the authors' knowledge, no study has prospectively investigated the measurement of vital signs, SIRS and qSOFA scores, and gut feeling in primary care patients with fever.

In this prospective study, it was investigated which vital signs were measured by GPs in OOH primary care patients with fever, and how often SIRS and qSOFA was calculated. The study investigated how many patients were referred to hospital, and whether vital signs, SIRS and qSOFA scores, and gut feeling were associated with referral. In addition, it was investigated how many patients were admitted to hospital (ward or intensive care unit [ICU]) and how many died within 30 days. It was expected that vital signs would not to be measured in all patients and the decision to refer would be multifactorial.

## Method

### Design and setting

In this prospective observational study, adult patients with fever (≥38.0°C) were included at two GP cooperatives (GPCs) in Heerlen and Maastricht, the Netherlands. In these GPCs, medical care is provided by GPs. Dutch GPs are obliged to work both in-hours and OOH. Patients who need medical care outside of office hours have to contact their nearby GPC first, after which nurses initially perform triage by telephone, using the computer-assisted triage method of the Netherlands Triage System. If physical assessment by a GP is deemed necessary, patients can get an appointment at the cooperative’s facility, or be scheduled for a home visit.^9^

The participating cooperatives in this study provide OOH primary care for 430 000 inhabitants of the region and are — as is customary in the Netherlands — located adjacent to the ED, which subsequently receive <3% walk-ins.^10^

The Strengthening the Reporting of Observational Studies in Epidemiology guidelines were used for reporting this observational study.^11^

### Patients

Patients eligible for inclusion were adults (aged ≥18 years) with a documented body temperature of ≥38.0°C (measured at home or by a GP), who were triaged to a face-to-face GP consultation at the GPC or a GP home visit. Refusal of written informed consent and a language barrier were exclusion criteria, as was a second presentation within the study period, since it is likely that these patients are treated differently. Owing to the labour-intensive design of the study, it was decided to include patients during two specific periods, mentioned below. A sample size calculation was not performed.

### Data collection

This study was conducted during two 9-day periods (1–9 September 2018 and 12–20 January 2019) including four weekend days from 08.00 to 23.30 hours and 10 weekdays from 17.00 to 23.30 hours. The GPs initially assessed and treated every eligible patient as they normally would. Immediately after the GP’s assessment, well-instructed research students included patients. As not all patients were expected to be included, age and sex of all eligible patients of one GPC were retrieved in order to investigate possible inclusion bias.

Immediately after inclusion, data on sex, age, duration of symptoms, and use of antibiotics during the current disease period were collected, as well as the following vital signs measured by the attending GP: blood pressure, heart rate, respiratory rate, temperature, and a Glasgow Coma Scale (GCS). Research students only measured missing vital signs required for SIRS criteria and qSOFA score calculations following a strict protocol. Blood pressure was measured electronically, heart and respiratory rate manually for 1 minute, temperature using an ear thermometer, and the GCS manually (normal or abnormal). Not considered essential for completeness were the two SIRS criteria leucocyte count and partial pressure of carbon dioxide (pCO_2_). In order to avoid influence of the study on the GP’s treatment plan, only abnormal measurements were reported back to the GP.

During the second 9-day period, the Gut Feelings Questionnaire (GFQ), a validated 10-item questionnaire, was used (see Supplementary Appendix S1).^7^ This questionnaire evaluates what level of a sense of alarm doctors feel when treating. GPs were asked to fill in the questionnaire after complete evaluation of the patient and after deciding whether or not the patient needed referral.

Follow-up data included hospital admission within 7 days and 30-day ICU admission and all-cause mortality.

### Analysis and statistics

Descriptive analyses were performed to evaluate the prevalence of fever, baseline characteristics, and the measurement of vital signs by GPs. The number of positive (≥2) SIRS and qSOFA scores was calculated using the GPs’ measurements alone and using all vital signs (after students had completed the additional measurements).

Next, the authors compared (the measurement of) vital signs by the GP and the number of positive SIRS and qSOFA scores (using all measurements performed) in patients who were referred and those who were not.

Descriptive analyses were performed regarding ED (re)visit, hospital and/or ICU admission, and mortality for all patients.

Statistical analyses were performed using IBM SPSS Statistics (version 26). Continuous data were reported as means with standard deviation (SD) and compared using Student's *t*-test, or as medians with interquartile ranges (IQR) and compared using the Mann-Whitney U test. Categorical data were reported as absolute numbers and as valid percentages; these were compared using χ^2^ or Fisher’s exact tests. A *P*-value <0.05 was considered statistically significant.

## Results

**Figure 1.**
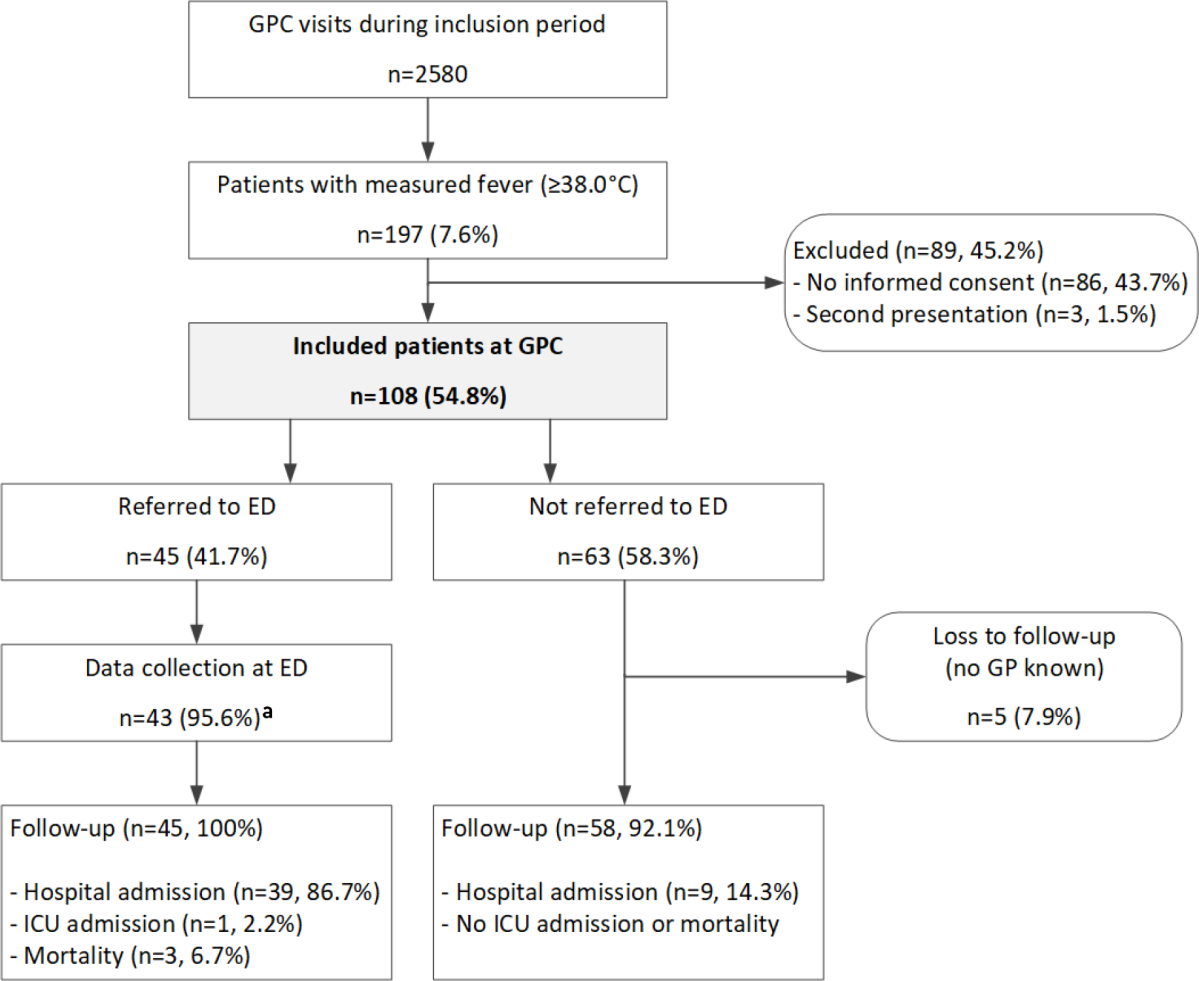
Flowchart of study population. ^a^Two patients were treated in a non-participating ED. GPC = GP cooperative. ED = emergency department. ICU = intensive care unit.

During the study period, 2580 adult patients visited the two GPCs (Figure 1). Of these, 197 had a temperature ≥38.0°C, resulting in a fever prevalence of 7.6%. Of these, 89 (45.2%) were excluded because of no informed consent (*n* = 86) or a second presentation in the study period (*n* = 3). In total, data of 108 (54.8%) patients were available for analysis. Excluded patients were younger than those included (median 58 years versus 72 years, *P*<0.001) (see Supplementary Table S1). There was no difference in sex between excluded and included patients (43.1% versus 39.7% male, *P* = 0.15).

### Baseline patient characteristics and vital sign measurements

Of 108 included patients, 38.0% (*n* = 41) were male and median age was 69 years (IQR 49–80 years) (Table 1). Median duration of symptoms was 1 day (IQR 0–3 days) and 13.9% (*n* = 15) of patients were already using antibiotics.

**Table 1.** Baseline characteristics and measurements of vital signs (*N* = 108)

**Baseline characteristics**	*n* (%)^a^			
Male	41 (38.0)			
Age, years, median (IQR)	69 (49–80)			
**Home visit**	61 (56.5)			
**Current disease period**				
Duration of symptoms, days, median (IQR)	1 (0–3)			
Use of antibiotics before GPC visit	15 (13.9)			
	**GP**		**GP + investigator**	
**Vital parameters**	**Measured**	**Median (IQR)**	**Measured**	**Median (IQR**)
Systolic blood pressure (mmHg)	94 (87.0)	130 (118–140)	107 (99.1)	130 (117–140)
Diastolic blood pressure (mmHg)	94 (87.0)	79 (70–81)	107 (99.1)	70 (70–81)
Heart rate (bpm)	87 (80.6)	97 (85–115)	106 (98.1)	95 (84–113)
Respiratory rate (breaths/min)	34 (31.5)	22 (18–28)	104 (96.3)	20 (18–26)
Temperature (°C)	99 (91.7)	38.5 (38.1–39.2)	107 (99.1)	38.5 (38.1–39.2)
Abnormal Glasgow Coma Scale, *n* (%)	71 (65.7)	17 (23.9)^b^	107 (99.1)	21 (19.6)^b^
**Clinical rules**				
Complete set of vital signs^c^	24 (22.2)		99 (91.7)	
All SIRS parameters measured	29 (26.9)		101 (93.5)	
SIRS ≥2	53 (49.1)		69 (63.9)	
All qSOFA parameters measured	27 (25.0)		102 (94.4)	
qSOFA ≥2	6 (5.6)		11 (10.2)	

^a^Values presented as *n* (%), unless stated otherwise. ^b^Number of patients with Glasgow Coma Scale (GCS) <15. ^c^Complete set of vital parameters = blood pressure, heart rate, respiratory rate, temperature, and GCS.

bpm = beats per minute. GPC = GP cooperative. qSOFA = quick Sequential Organ Failure Assessment. SIRS = systemic inflammatory response syndrome.

GPs measured a complete set of vital signs required for SIRS criteria and qSOFA score calculations in 24 (22.2%) patients. The respiratory rate was measured least (*n* = 34, 31.5%), while the temperature was measured most frequently (*n* = 99, 91.7%). Positive (≥2) SIRS scores were present in 53 (49.1%) patients and positive (≥2) qSOFA scores in 6 (5.6%) patients, based on the GPs’ measurements. These numbers increased to 69 (63.9%) for SIRS and 11 (10.2%) for qSOFA after adding missing vital signs.

In total, researchers performed 146 additional measurements. Of these, 42 (28.8%) were abnormal (mostly the respiratory rate [76.2%]) and therefore reported back to the GP (Table 2). Not once did these abnormal findings change the decision whether or not to refer a patient.

**Table 2.** Abnormal vital signs reported back to GPs

**Vital sign**	**Cut-off value for back-reporting to GP**	**Reported back to treating GP, *n***	**Referred to ED, *n* (%**)	**Change in referral strategy, *n***
Systolic blood pressure	<90 mmHg	0	—	—
Heart rate	>100 bpm	4	3 (75.0)	0
Respiratory rate	>20/min	32	17 (53.1)	0
Glasgow Coma Scale	<15	4	3 (75.0)	0
Temperature	>40°C	0	—	—

bpm = beats per minute. ED = emergency department.

### Comparison between referred patients and patients who were not referred

GPs referred 45 (41.7%) patients to the ED (Table 3). These patients had higher respiratory rates (24 versus 18, *P*<0.001) and temperatures (38.6°C versus 38.3°C, *P* = 0.02) and more often an abnormal GCS (31.1% versus 11.3%, *P* = 0.01) than patients who were not referred. Positive SIRS and qSOFA scores were more prevalent in the referred group (75.6% versus 55.6%, *P* = 0.03 and 22.2% versus 1.6%, *P* = 0.001, respectively), as was a sense of alarm (76.5% versus 14.7%, *P*<0.001). Of the patients who were not referred, 35 (55.6%) had SIRS ≥2 and one (1.6%) qSOFA ≥2.

**Table 3.** Comparison between patients who were referred and patients who were not referred

	Patients who were referred	Patients who were not referred
*n*	Value^a^	Value^a^	*P* value
**Baseline characteristics**	45	—	63	—
Male	108	19 (42.2)	22 (34.9)	0.44
Age, years, median (IQR)	108	69 (56–81)	65 (40–79)	0.16
Home visit	108	29 (64.4)	32 (50.8)	0.16
**Vital signs** **at GPC**	45	—	63	—
Systolic blood pressure, mmHg, median (IQR)	107	128 (110–140)	130 (120–140)	0.26
Heart rate, bpm, median (IQR)	106	100 (87.8–120.0)	95 (83.5–104.3)	0.15
Respiratory rate, /min, median (IQR)	104	24 (19–30)	18 (16–24)	<0.001
Temperature, °C, median (IQR)	107	38.6 (38.2–39.4)	38.3 (38.0–39.3)	0.02
Glasgow Comma Scale <15	107	14 (31.1)	7 (11.3)	0.01
Complete set of vital signs measured by GP	108	13 (28.9)	11 (17.5)	0.16
**Clinical rules**	45	—	63	—
SIRS ≥2	108	34 (75.6)	35 (55.6)	0.03
qSOFA ≥2	108	10 (22.2)	1 (1.6)	0.001
**Gut feeling**	17	—	34	—
Sense of alarm present	51^b^	13 (76.5)	5 (14.7)	<0.001

^a^Values presented as *n* (%), unless otherwise stated. ^b^Gut feeling questionnaires were only done during the second inclusion period of the study.

bpm = beats per minute. GPC = GP cooperative. qSOFA = quick Sequential Organ Failure Assessment. SIRS = systemic inflammatory response syndrome.

### Follow-up

In total, one (2.2%) patient was admitted to ICU and three (6.7%) patients died within 30 days (Figure 1). All of these patients were primarily referred to the ED. Nine (14.3%) of 63 patients who were initially not referred were admitted to hospital within 1 week after the GPC visit. Six (66.7%) of these nine patients already had a positive SIRS score based solely on the GP’s measurements, while none were qSOFA positive (Supplemental Table 1).

## Discussion

### Summary

In the study, GPs measured a complete set of vital signs for SIRS criteria and qSOFA score calculations in only 22.2% of patients. With their measurements, GPs could have calculated positive SIRS and qSOFA scores in 49.1% and 5.6% of patients, respectively. After missing vital signs were measured, these numbers increased to 63.9% and 10.2%. Patients who were referred had higher respiratory rates and temperatures, and more often an abnormal GCS than patients who were not referred. In addition, positive SIRS and qSOFA scores and a sense of alarm were more prevalent in patients who were referred. Of patients who were not referred, 14.3% were admitted to hospital (ward) within 7 days of the consultation. ICU admission and mortality happened in patients who were referred only.

### Strengths and limitations

To the authors’ knowledge, this is the first prospective study investigating the measurement of vital signs, gut feeling, and referral strategies of primary care patients with fever. The unique study design allowed the authors to investigate the real-world approach of GPs and still use complete sets of vital signs. This resulted in almost no missing vital signs and complete follow-up. Both summer and winter were included to consider seasonal differences. Nonetheless, an important limitation of the labour-intensive design is the achieved sample size. Despite placing maximum effort on patient inclusion, 89 patients were not included mainly owing to refusing informed consent (*n* = 86). The fact that included patients were older than excluded patients may have inflated referral rates. More precise information to investigate possible selection bias was not available. Owing to these limitations, it is concluded that this study must be seen as a pilot study that justifies further and larger investigations into the decision-making process of GPs in patients with fever. In these studies, moving consent and measurement of the vital signs forward (that is, to the waiting time) could result in partial improvement of the inclusion rates.

### Comparison with existing literature

In this study, GPs measured a complete set of vital signs in only one-fifth of patients. The respiratory rate was measured least (31.5%) and less often than in other studies, but blood pressure, heart rate, and temperature were measured more frequently.^6,12^ A possible explanation lies in the study design. Although GPs were asked to perform their work-up as they normally would, they may have measured more vital signs than usual owing to the presence of research students (Hawthorne effect).^13^ This overestimation might also have occurred in other studies, as GPs were asked to recall their last-referred patient with a serious infection, causing possible recall bias.^6,12^ How GPs decide if and which vital signs they measure in a patient is not known, but may depend on habit or ritual, or even a patient’s expectations. In addition, GPs’ association of some vital signs with disease severity may play a role (for example, blood pressure in relation to shock). One could argue that GPs sometimes decide to refer a patient on the basis of one or two vital signs, and measuring the others is therefore redundant. The authors, however, would like to argue against this. Although measuring all vital parameters may not necessarily affect the referral decision, complete information on a patient’s current vital sign status is indispensable for identifying trends and urgency throughout the acute care chain.

The respiratory rate was measured in only a minority of patients. Explanations include the fact that the respiratory rate is mostly measured manually and the belief measuring it wastes valuable time.^14–19^ Not only was it measured least, but also the respiratory rate was most frequently reported back to the GP after it was found to be abnormal by the research students (*n* = 32). Not once did this change the decision whether or not to refer the patient; almost half (46.9%) of these patients were not referred to the ED. The most likely explanation is that the respiratory rate does not solely determine the decision to refer. Some patients may always have an elevated respiratory rate or their other vital signs are reassuring. Another possibility is that GPs underestimate the prognostic value of an elevated respiratory rate, but the extensive attention this specific vital sign has received over the past years makes this less likely. Finally, the cut-off point of >20/min could be inadequate for primary care settings. It is likely that GPs assess many patients with an elevated respiratory rate who recover without complications, making them less aware of mild elevations. The true value of the respiratory rate in primary care settings can only be determined once accurately measured in all patients.

Another important finding is that 35 patients with positive SIRS scores and one patient with a positive qSOFA score were not referred to the ED. In general, this turned out to be a safe approach, as none of these were admitted to the ICU or died. However, 14.3% of the initally not referred patients were admitted to the hospital within 7 days of the primary consultation. In a recent survey, GPs’ knowledge of the SIRS criteria was substantially higher than of the qSOFA.^20^ It is therefore somewhat surprising that the majority (66.7%) of patients who were admitted to the hospital within 7 days after the inital GPC visit had a positive SIRS score using the vital signs initially measured by the GP (see Supplementary Table S2). This shows that positive SIRS scores do not automatically trigger a specific assessment or treatment protocol. The probability of finding a patient with sepsis differs substantially between the primary care and hospital setting. Since GPs do not measure all vital signs needed to calculate these scores and not all patients with positive scores are referred, it is likely that SIRS and qSOFA scores alone do not determine the decision to refer a patient with fever to the ED.

To further investigate GPs’ referral strategies, GPs were asked to complete the GFQ.^7^ Like others, an association was found between the presence of a sense of alarm and referral.^7,8^ Five (14.7%) of the patients who were not referred raised a sense of alarm. A possible explanation may be that the GFQ does specify why there is a sense of alarm, which could be caused, for example, by the suspicion of an underlying malignancy. As with vital signs, referral decisions could not be explained by gut feeling alone.

### Implications for research and practice

In the present study, GPs measured a complete set of vital signs in one-in-five patients with fever. Although associations were found between some vital signs, clinical rules, gut feeling, and ED referral, the decision to refer a patient is probably multifactorial. Future research may focus on the diagnostic and prognostic value of vital signs, the use of point-of-care tests, such as lactate and/or C-reactive protein, and gut feeling in primary care to help develop diagnostic algorithms for GPs that aid in the decision to refer. For these algorithms to work adequately, systematic measurement and recording of vital signs is indispensable, either manually or by using electronic devices.
